# Plasma sRAGE levels strongly associate with centrilobular emphysema assessed by HRCT scans

**DOI:** 10.1186/s12931-022-01934-w

**Published:** 2022-01-24

**Authors:** Frank Klont, Peter Horvatovich, Russell P. Bowler, Eva van Rikxoort, Jean-Paul Charbonnier, Marcel Kwiatkowski, David A. Lynch, Stephen Humphries, Rainer Bischoff, Nick H. T. ten Hacken, Simon D. Pouwels

**Affiliations:** 1 Department of Analytical Biochemistry, Groningen Research Institute of Pharmacy, University of Groningen, Antonius Deusinglaan 1, 9713 AV Groningen, The Netherlands; 2 Groningen Research Institute for Asthma and COPD (GRIAC), University Medical Center Groningen, Groningen, The Netherlands; 3 Department of Medicine, National Jewish Health, Denver, USA; 4 Thirona, Nijmegen, The Netherlands; 5 Diagnostic Image Analysis Group, Department of Radiology, Radboud University Nijmegen Medical Center, Nijmegen, The Netherlands; 6 Department of Radiology, National Jewish Health, 1400 Jackson St, Denver, CO 80206 USA; 7 Department of Pulmonary Diseases, University Medical Center Groningen, Hanzeplein 1, 9713 GZ Groningen, The Netherlands; 8 Department of Pathology and Medical Biology, University Medical Center Groningen, Groningen, The Netherlands

**Keywords:** COPD biomarkers, Emphysema, CT phenotyping, sRAGE

## Abstract

**Background:**

There is a strong need for biomarkers to better characterize individuals with COPD and to take into account the heterogeneity of COPD. The blood protein sRAGE has been put forward as promising biomarker for COPD in general and emphysema in particular. Here, we measured plasma sRAGE levels using quantitative LC–MS and assessed whether the plasma sRAGE levels associate with (changes in) lung function, radiological emphysema parameters, and radiological subtypes of emphysema.

**Methods:**

Three hundred and twenty-four COPD patients (mean FEV_1_: 63%predicted) and 185 healthy controls from the COPDGene study were selected. Plasma sRAGE was measured by immunoprecipitation in 96-well plate methodology to enrich sRAGE, followed by targeted quantitative liquid chromatography-mass spectrometry. Spirometry and HRCT scans (inspiration and expiration) with a 5-year follow-up were used; both subjected to high quality control standards.

**Results:**

Lower sRAGE values significantly associated with the presence of COPD, the severity of airflow obstruction, the severity of emphysema on HRCT, the heterogeneous distribution of emphysema, centrilobular emphysema, and 5-year progression of emphysema. However, sRAGE values did not associate with airway wall thickness or paraseptal emphysema.

**Conclusions:**

Rather than being a general COPD biomarker, sRAGE is especially a promising biomarker for centrilobular emphysema. Follow-up studies should elucidate whether sRAGE can be used as a biomarker for other COPD phenotypes as well.

## Background

Chronic obstructive pulmonary disease (COPD) is characterized by persistent airflow limitation that is usually progressive and associates with an enhanced chronic airway inflammatory response in response to noxious particles or gases. The forced expiratory volume in one second (FEV_1_)/forced vital capacity (FVC) is used for the diagnosis and the FEV_1_ percent predicted is used for staging of this disease, however lung function does not provide information regarding disease activity or the underlying pathologic processes, cannot distinguish the various phenotypes of COPD such as emphysema, and is not specific for COPD. Novel biomarkers for COPD have therefore been explored in large-scale longitudinal studies like ECLIPSE, SUMMIT, SPIROMICS and COPDgene [1–5].

All these studies identified the soluble receptor for advanced glycation end-products (sRAGE) to be amongst the most promising blood biomarkers. sRAGE is the extracellular domain of the pro-inflammatory pattern recognition receptor RAGE. The freely circulating soluble form of RAGE can either be produced by alternative splicing of the *AGER* gene or by proteolytic cleavage of the receptor by proteases like MMP9 and ADAM10 [6]. While the membrane bound receptor has pro-inflammatory functions, sRAGE has anti-inflammatory properties by acting as a decoy receptor as well as preventing the homodimerization of RAGE, needed for down-stream signaling [7]. Recently, it became evident that RAGE signaling plays a key role in the development of COPD, contributing both to airway inflammation as well as emphysema [8–11]. Furthermore, a genetic polymorphism, rs2070600, within the *AGER* locus was found to be associated with circulating sRAGE levels and the risk of COPD development [12, 13]. Very robustly, all studies investigating sRAGE levels in COPD patients have found lower sRAGE levels in COPD patients [14–17] and found sRAGE to be associated with neutrophilic airway inflammation [18], acute exacerbation [17], decline in FEV_1_ [16], and presence of emphysema [19]. Particularly the association with emphysema is strong, showing significant correlations with carbon monoxide (CO) diffusion capacity [13, 20], and a number of quantitative HRCT measurements that reflect emphysema [20–22], as well as progression of emphysema [23].

Recently, we developed a targeted quantitative liquid chromatography-mass spectrometry (LC–MS)-based assay to detect and quantify sRAGE in blood [24]. This measurement is fully validated according to the U.S. Food and Drug Administration and European Medicines Agency guidelines and demonstrated decreased values in COPD patients, correlating with lung function and autofluorescence of advanced glycation end-products in the skin [15]. Furthermore, using this newly developed sRAGE assay, we found that smoking strongly decreases the serum levels of sRAGE within 2 h [25]. To further determine the value of sRAGE as biomarker for COPD, we now assessed the plasma sRAGE levels in the large, clinically well-characterized multi-center COPDGene cohort. The COPDGene study was chosen because we aimed to determine the role of sRAGE as a biomarker in subtypes of emphysema and the progression of emphysema over time, which could be assessed in COPDGene because this study had a 5-year follow-up, and because COPD patients were radiologically phenotyped on HRCT using a standardized classification [26].

## Methods

### Study participants

The study was approved by the Institutional Review Board of the National Jewish Health and all participants gave informed written consent. Former and current smokers with COPD and former and current smokers without spirometric impairment (called GOLD 0) were selected from the COPDGene study based on matching of age and body mass index (BMI) (Table 1). The GOLD 0 subject group has previously been defined as current and former smokers with a normal postbronchodilator ratio of FEV_1_ to forced vital capacity exceeding 0.7 and a FEV_1_ percentage of at least 80% predicted [27]. Subjects were between 45 and 80 years of age with a minimum of 10 pack-years smoking history (except non-smoking controls) [26]. In the COPDGene study, COPD was defined by a post-bronchodilator forced expiratory volume in the first second (FEV_1_) to forced vital capacity (FVC) ratio of < 0.70. Plasma was obtained from a venipuncture and P100 tube during the first (enrolling) visit on the same day as spirometry and CT scan.

**Table 1:** Subject characteristics

	COPD patients	Healthy controls (GOLD 0)	p-values
Number of subjects	324	185	NA
Number male subjects, %	170 (52%)	75 (41%)	**0.0100**
Age, years	64.9 (8.0)	65.0 (5.8)	0.94
BMI, kg/m^2^	28.4 (5.5)	29.3 (4.9)	0.07
Number current smoker, %	84 (26%)	45 (24%)	0.75
Pack years	51.4 (25.0)	39.3 (22.0)	** < 0.0001**
GOLD stages (I/II/III/IV)	79/144/78/23	NA	NA
FEV_1_, % predicted	62.4 (22.1)	98.0 (11.2)	** < 0.0001**
Decrease in FEV_1_ in 5 years, mL	209 (281)	218 (215)	0.70

Data presented as mean (SD) or number (% proportion), statistical significant results are depicted in bold

*NA* not applicable

### Measurements

sRAGE was measured at baseline during the first visit of the COPDgene study. sRAGE was measured using a simplified immunoprecipitation in 96-well ELISA (IPE) methodology to enrich sRAGE coupled to targeted liquid chromatography-mass spectrometry (LC–MS) [24]. This assay measures sRAGE at clinically relevant levels between 0.1 and 10 ng/mL, which necessitates a considerable degree of sample cleanup for which anti-sRAGE antibodies (R&D Systems, Cat. No. MAB11451, clone 176902) were used. LC–MS analyses were performed with a Waters Ionkey/MS system using an ACQUITY M-Class UPLC and a XEVO mass spectrometer (Milford, MA, U.S.A.), and sRAGE was detected based on two protein-specific tryptic peptides (i.e., IGEPLVLK, VLSPQGGGPWDSVAR) originating from sRAGE’s N-terminal region which is essential for binding to most of its ligands. sRAGE values demonstrated a normal distribution in our population.

HRCT-scans were acquired using multi-detector CT scanners with at least 16 detector channels [26]. Volumetric CT acquisitions were obtained both on full inspiration (200 mAs), and at the end of normal expiration (50 mAs). Image reconstruction utilized sub-millimeter slice thickness, with smooth and edge-enhancing algorithms [26].

CT phenotyping was performed on segmented lung images, using Thirona Lung Quantification software (Thirona, http://www.thirona.eu). Total inspiratory and expiratory lung volumes, mean lung attenuation, and percentage of low attenuation areas (%LAA) below -950HU on inspiration and below -856HU on expiration were determined for the whole lung and each of the lobes independently [26]. Presence of emphysema was considered if %LAA below -950HU was larger than 6% [28], presence of heterogeneous emphysema was considered if the difference in %LAA below − 950HU between the left upper lobe (LUL) and left lower lobe (LLL) or between the right upper lobe (RUL) plus middle lobe (RML) and right lower lobe (RLL) was larger than 10%. Additionally, the 15th percentile of the lung density histogram within patients was determined (Perc15) [29]. Airway wall thickness was expressed as Pi10, defined as the square root of the wall area at the inner perimeter of a 10 mm diameter airway. This measurement provides a useful summary score of the airway wall thickness for an individual patient, and has shown to be a measure for smoking-related airway injury that can provide important information regarding longitudinal changes in airway wall thickness [30, 31].

Parallel imaging analyses of percent emphysema and percent gas trapping were performed using 3D Slicer (http://www.slicer.org/). Parametric Response Mapping (PRM) provided 3 categories: emphysema (all voxels below -950HU in the inspiratory CT and below -856 HU in the expiratory CT), air trapping (all voxels above -950HU in the inspiratory CT and below -856 HU in the expiratory CT), and normal (all voxels above both thresholds in both scans) [32]. Paraseptal emphysema was visually scored as absent, mild or substantial, and centrilobular emphysema was scored as absent, trace, mild, moderate, confluent or advanced destructive as previously described [33].

All spirometry data were collected using an EasyOne spirometer (ndd Medical Technologies, Zurich, Switzerland) and were reviewed by the pulmonary function test quality assurance core analyst of the COPDGene Study [34]. Spirometric data were typically collected at the same day as the acquired CT studies (mean time between spirometry and CT, 0.31 h).

### Statistics

Data evaluation was performed using SPSS statistical software package version 23.0. p-values < 0.05 were considered statistically significant. Differences in clinical variables (age, pack years, BMI, lung function) and quantitative HRCT measures (volumes, mean densities, %LAA, Pi10, Perc15, and PRM parameters) between COPD patients and GOLD 0 were analyzed by T-Test or Mann–Whitney U, and differences between GOLD stages by Kruskal–Wallis tests. Categorical variables (sex, current smokers, centrilobular emphysema, paraseptal emphysema) were analyzed by a Chi-square test. Correlations between Srage and clinical or quantitative HRCT measures were examined by Pearson’s or Spearman’s Rank tests. The independent contribution of Srage to the variation of (5-year changes in) FEV_1_ and (5-year changes in) quantitative HRCT measures were analyzed by multiple linear regression adjusted for age, gender, current smoking, pack years, and BMI.

## Results

### Subject characteristics

Three hundred and twenty four COPD patients and 185 healthy controls (GOLD 0) were selected from the COPDGene cohort. Patient characteristics are described in Table 1. Within the COPD group, 26% were current smokers and within the healthy control group, 24% of the individuals were current smokers. Males were significantly more present in the COPD group as compared to controls: 52% vs 41% (p = 0.0100). The mean (SD) FEV_1_ of the COPD group was 62.4 (22.1) %predicted, indicating mild to moderate severe disease. Mean annual decline in FEV_1_ was similar between COPD patients and GOLD 0 controls: 42 vs 44 mL/year.

### sRAGE correlation with COPD characteristics

**Fig. 1:**
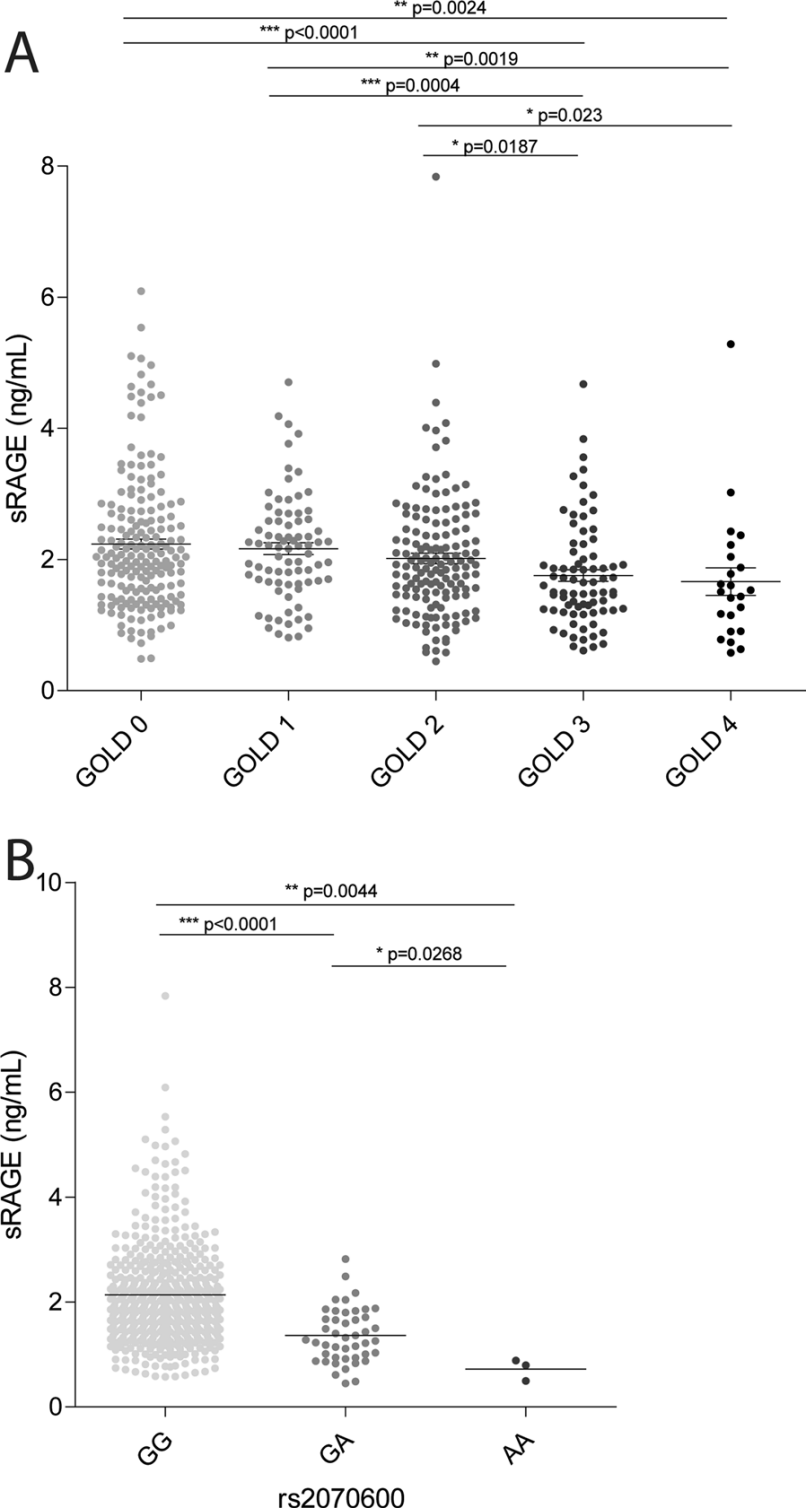
sRAGE in healthy controls (GOLD 0) and COPD patients (GOLD 1–4). **A** The levels of sRAGE were assessed in plasma using the simplified immunoprecipitation in 96-well ELISA (IPE) methodology coupled to targeted liquid chromatography-mass spectrometry (LC–MS). Plasma sRAGE levels were assessed in 185 healthy individuals without airway obstruction (GOLD 0), 79 GOLD stage I COPD patients, 144 GOLD stage II COPD patients, 78 GOLD stage III COPD patients and 23 GOLD stage IV COPD patients. **b** The plasma levels of sRAGE in subjects homozygous for the major allele of rs2070600 (GG), heterozygous (GA), or homozygous for the minor allele of rs2070600 (AA). Data is shown as individuals data points and mean ± SEM. Statistical differences were tested using a Mann–Whitney U test, *p < 0.05, **p < 0.01, ***p < 0.001. The exact p-values are indicated where appropriate

Mean (SD) sRAGE levels were significantly lower in COPD compared to GOLD 0 controls: 1.97 (0.90) vs 2.23 (1.03) ng/mL, and more severe COPD stages demonstrated lower values compared to milder COPD stages (Fig. 1a). There was a weak but significant correlation between sRAGE and FEV_1_%predicted in the total and COPD population: R = 0.23 (p < 0.0001) and 0.25 (p < 0.0001), respectively. Significant but weaker correlations were also found for FEV1 (0.15, p = 0.0007) and FEV1/FVC (0.19, p < 0.0001) in the total population and for FEV1 (0.17, p = 0.0025) and FEV1/FVC (0.21, p = 0.0001) in the COPD population. In a multiple-linear regression analysis, sRAGE independently contributed to FEV_1_ (Table 2). sRAGE did not associate with 5-year change in FEV_1_, neither in ordinary nor in multiple-linear regression analyses. Additionally, we showed that the levels of sRAGE are significantly lower in subjects possessing the minor allele of the AGER polymorphism rs2070600 (Fig. 1b). This decrease was most severe in homozygotes for the minor allele (AA), but was already present in heterozygotes (GA).

**Table 2:** sRAGE explaining the variation in FEV1 and quantitative and visual HRCT measurements

*Baseline*										
	FEV1		%LAA Below -950HU		Centrilobular emphysema		Pi10		Air trapping (PRM)	
	R square: 0.199		R square: 0.235		R square: 0.280		R square: 0.090		R square: 0.186	
	Beta	p-value	Beta	p-value	Beta	p-value	Beta	p-value	Beta	p-value
sRAGE	0.159	**0.0001**	− 0.229	** < 0.0001**	0.188	** < 0.0001**	− 0.046	0.31	− 0.143	**0.0009**
Sex	− 0.379	** < 0.0001**	− 0.143	**0.0005**	− 0.059	0.14	− 0.055	0.22	− 0.159	**0.0002**
Age	− 0.139	**0.0017**	− 0.041	0.34	0.014	0.74	− 0.118	**0.0135**	0.120	**0.0093**
BMI	0.020	0.62	− 0.323	** < 0.0001**	− 0.323	** < 0.0001**	0.120	**0.0062**	− 0.218	** < 0.0001**
Current smoking	0.067	0.11	− 0.238	** < 0.0001**	− 0.090	**0.0280**	0.102	**0.0269**	− 0.161	**0.0003**
Pack years	− 0.220	** < 0.0001**	0.183	** < 0.0001**	0.387	** < 0.0001**	0.187	** < 0.0001**	0.195	** < 0.0001**

Change between baseline and 5-year follow-up										
	FEV1		%LAA Below -950HU		Centrilobular emphysema		Pi10		Air trapping (PRM)	
	R square: 0.051		R square: 0.103		R square:		R square: 0.013		R square: 0.051	
	Beta	p-value	Beta	p-value	Beta	p-value	Beta	p-value	Beta	p-value
sRAGE	0.020	0.66	− 0.172	**0.0003**	ND	ND	− 0.033	0.50	− 0.002	0.97
Sex	− 0.204	** < 0.0001**	− 0.084	0.08	ND	ND	− 0.072	0.15	0.007	0.89
Age	− 0.055	0.26	− 0.060	0.23	ND	ND	0.033	0.53	− 0.108	**0.0433**
BMI	− 0.085	0.05	− 0.146	**0.0019**	ND	ND	0.037	0.46	− 0.068	0.17
Curent smoking	0.030	0.52	0.150	**0.0024**	ND	ND	0.014	0.79	0.142	**0.0061**
Pack years	− 0.079	0.08	0.098	**0.0400**	ND	ND	− 0.084	0.09	0.063	0.21

*Beta* standardized coefficient. Adjusted for age at enrollment, sex, BMI, current smoking, pack years. *ND* not determined (no 5-year follow-up scores done). sRAGE levels, BMI, smoking status and pack years were obtained at baseline, lung function parameters were obtained at baseline and at 5-year follow-up, statistical significant results are depicted in bold

### HRCT analyses

Quantitative HRCT analyses demonstrated lower lung attenuation and higher airway wall thickness in COPD compared to GOLD 0 controls (Table 3). The proportion of individuals with radiological emphysema, based on %LAA below -950HU ≥ 6%, was higher in COPD patients than in GOLD 0 controls: 51% vs 11%, respectively (p < 0.0001). Within the group of individuals with %LAA below − 950HU ≥ 6% heterogeneous emphysema was present in 30% and homogeneous emphysema in 70% of the individuals (Table 4). Visually scored centrilobular emphysema (score > 0) was present in 86% of the COPD patients and 47% in the GOLD 0 controls (p < 0.0001). Paraseptal emphysema (> score 0) was present in 46% of the COPD patients and 29% of the GOLD 0 controls (p < 0.0001). The 5-year change in emphysema assessed by %LAA, Perc15 and PRM parameters was significantly higher in COPD patients than GOLD 0 controls (Table 3). In contrast, the 5-year change in airway wall thickness and air trapping was not significantly different between these two groups.

**Table 3:** HRCT characteristics and correlation with sRAGE

	Radiological difference between healthy and COPD			sRAGE correlation within total group	
	COPD patients	Healthy controls (GOLD 0)	P-value	Rho	P-value
LAA below -950 HU, %	10.37 (10.59)	2.41 (3.00)	** < 0.0001**	− 0.241	** < 0.0001**
*5-year change*	1.83 (4.85)	− 0.36 (2.38)	** < 0.0001**	− 0.187	**0.0001**
LAA % below -856 HU, %	30.85 (18.77)	10.08 (7.94)	** < 0.0001**	− 0.186	** < 0.0001**
*5-year change*	3.29 (9.83)	0.75 (6.20)	**0.0036**	− 0.115	**0.0183**
Perc15, HU	934.6 (22.8)	914.8 (17.9)	** < 0.0001**	0.201	** < 0.0001**
*5-year change*	− 3.65 (10.66)	0.71 (13.06)	**0.0001**	0.082	0.09
PRM Emphysema, %	8.9 (10.5)	0.9 (1.5)	** < 0.0001**	− 0.226	** < 0.0001**
*5-year change*	2.24 (4.85)	0.03 (1.1)	** < 0.0001**	− 0.217	** < 0.0001**
PRM Air trapping, %	22.9 (12.2)	9.2 (6.5)	** < 0.0001**	− 0.147	**0.0014**
*5-year change*	1.43 (7.64)	0.87 (5.00)	0.41	− 0.015	0.75
PRM Normal, %	65.7 (20.5)	88.3 (8.8)	** < 0.0001**	0.210	** < 0.0001**
*5-year change*	− 3.45 (9.91)	− 0.54 (6.65)	**0.0109**	0.109	**0.0254**
Pi10, mm	2.43 (0.52)	1.85 (0.34)	** < 0.0001**	− 0.099	**0.0270**
*5-year change*	0.03 (0.33)	0.03 (0.14)	0.96	− 0.033	0.49
Centrilobular emphysema	2 (0–5)	0 (0–3)	** < 0.0001**	− 0.197	** < 0.0001**
*5-year change*	NA	NA	NA	NA	NA
Paraseptal emphysema	0 (0–2)	0 (0–2)	** < 0.0001**	− 0.081	0.07
*5-year change*	NA	NA	NA	NA	NA

Group data given in mean (standard deviation), or median (range)

*LAA* low attenuation area, *Perc15* 15th percentile of the lung density histogram (HU), *Pi10* airway wall thickness at an internal perimeter of 10 mm, *PRM* parametric response mapping, *NA* not available, statistical significant results are depicted in bold

**Table 4:** sRAGE in different subtypes of emphysema

	No Emphysema (LAA below − 950HU < 6%)	Emphysema (LAA below -950HU $\ge$ 6%)		
		Homogeneous	Heterogeneous	
		No dominance − 10% < HD < 10%	Upper HD > 10%	Lower HD < − 10%
Number	315	125	45	9
Presence COPD	49%^1^	86%^2a^	98%	100%
FEV_1_%pred	84.7% (20.0)^1^	63.1% (26.5)^2a^	56.9% (24.3)^3^	36.6% (10.3)
%LAA below -950HU	1.97 (1.65)^1^	13.86 (7.65)^2c^	23.38 (9.65)	29.19 (11.16)
Centrilobular	1 (0–4)^1^	3 (0–5) ^2c^	4 (3–5)^3^	4 (2–5)
Paraseptal	0 (0–2)^1^	0 (0–2)^2a^	1 (0–2)	0 (0–2)
sRAGE	2.22 (1.02)^1^	1.89 (0.75)^2b^	1.53 (0.73)	1.57 (0.45)

Data given in number, percentage, mean (standard deviation), or median (range). HD: heterogeneity difference (Upper–Lower Lobes %LAA below -950HU)

^1^p < 0.0001 vs emphysema

^2a^p < 0.05 vs heterogeneous emphysema

^2b^p < 0.01 vs heterogeneous emphysema

^2c^p < 0.0001 vs heterogeneous emphysema

^3^p < 0.05 vs lower lobe heterogeneous emphysema

### sRAGE associations with HRCT

**Fig. 2:**
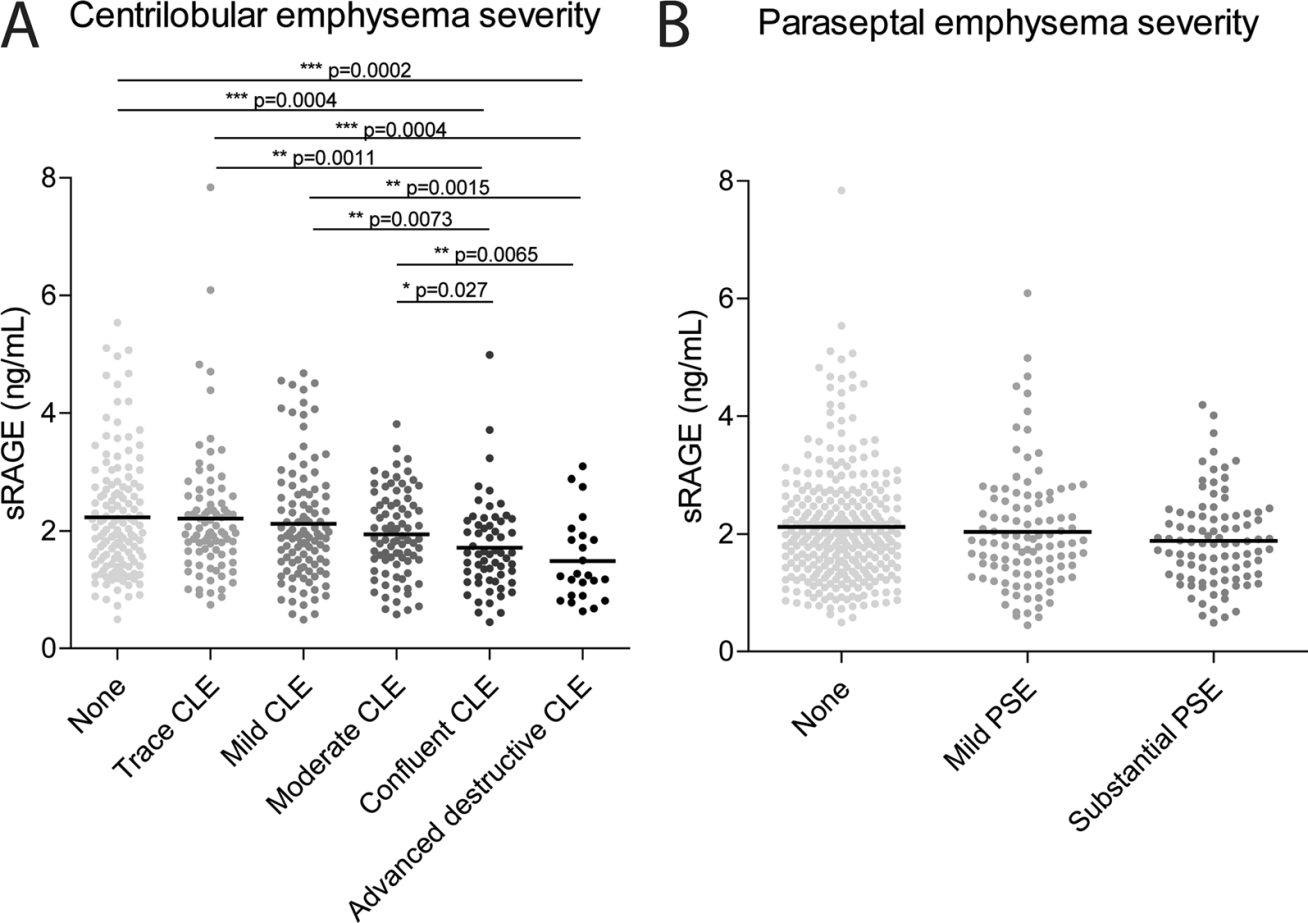
sRAGE associates with centrilobular emphysema. The levels of sRAGE were assessed in plasma using the simplified immunoprecipitation in 96-well ELISA (IPE) methodology coupled to targeted liquid chromatography-mass spectrometry (LC–MS). **A** The association of plasma sRAGE with centrilobular emphysema (CLE) was assessed by measuring sRAGE in plasma of 139 subjects without CLE, 85 subjects with trace CLE, 107 subjects with mild CLE, 82 subjects with moderate CLE, 60 subjects with confluent CLE and 24 subjects with advanced destructive CLE. **B** The association of plasma sRAGE with paraseptal emphysema (PSE) was assessed by measuring sRAGE in plasma of 299 subjects without PSE, 105 subjects with mild PSE and 93 subjects with substantial PSE. Data is shown as individuals data points and mean ± SEM. Statistical differences were tested using a Mann–Whitney U test, *p < 0.05, **p < 0.01, ***p < 0.001. The exact p-values are indicated where appropriate

sRAGE correlated significantly with all quantitative HRCT measurements (Table 2). In multiple-linear regression analysis, lower sRAGE levels contributed independently to higher emphysema scores based on %LAA below 950HU (Table 4), and higher air trapping scores based on PRM scores (Table 4). However, there was no significant association with airway wall thickness assessed by Pi10. sRAGE levels were significantly lower in heterogeneous compared to homogeneous emphysema, but there was no difference between upper lobe versus lower lobe predominant emphysema (Table 3). sRAGE levels were significantly lower in more severe centrilobular emphysema (Fig. 2), but there was no association with paraseptal emphysema. In 5-year follow-up, lower sRAGE levels correlated significantly with changes in most emphysema parameters, but not with airway wall thickness, nor air trapping (Table 2). In a multiple-linear regression analysis, lower sRAGE values contributed independently to the 5-year progression of emphysema, but not to the change in airway thickness, nor air trapping (Table 4).

## Discussion

We found that lower plasma sRAGE levels were significantly associated with the presence of COPD, the AGER polymorphism rs2070600, the severity of airflow obstruction, the severity of emphysema on HRCT, heterogeneous distribution of emphysema, the centrilobular subtype of emphysema, and 5-year progression of emphysema severity. In contrast, sRAGE levels did not associate with airway wall thickness or the paraseptal subtype of emphysema. As the highest associations were observed with the severity of centrilobular emphysema, sRAGE seems to be an attractive biomarker for this radiological subtype of emphysema.

A novel finding of this study is the strong association of sRAGE with centrilobular emphysema, the most common form of smoking-related emphysema which involves lung tissue around the terminal bronchioles, at the center of the secondary lobules [35]. sRAGE demonstrated a significant association with heterogeneous emphysema, but not specifically with upper or lower lung predominance. It is unclear whether sRAGE independently contributes to the presence of heterogeneous emphysema, as heterogeneous emphysema (according to our definition) can only be present in more severely emphysematous lungs. An important additional finding of our study is the absence of a relationship with paraseptal emphysema, contributing to the idea that sRAGE is a more specific COPD sub-type biomarker. This finding is not unexpected, taking into account the completely different underlying pathology of centrilobular and paraseptal emphysema [28], whereas centrilobular emphysema is characterized by destruction of the central parts of the lung lobule, paraseptal emphysema is characterized by destruction of the lung lobules near the lobular septa and is observed mainly near the pleural surface in the upper regions of the lungs.

Because we were primarily interested in the biomarker function of plasma sRAGE in COPD and not in smoking-related effects, we did not compare COPD patients to never smokers but to a previously defined GOLD 0 group. This group encompasses former and current smokers that do not display any spirometric impairment. Our study confirms earlier reports suggesting that sRAGE shows a higher association with emphysema compared to chronic bronchitis [13, 19]. In this regard, sRAGE did not associate with Pi10, a marker reflecting airway wall thickness and associated with symptom-based chronic bronchitis [36]. Our study demonstrated also a significant association between sRAGE and FEV_1_, however this association was weaker compared to the association between sRAGE and emphysema. From literature it was known that emphysema independently contributes to airflow obstruction reflected by FEV_1_ and FEV_1_/FVC, a contribution that is relatively more important than that of airway wall thickness [37]. We hypothesized that sRAGE contributes indirectly to FEV_1,_ via emphysema, and not airway wall thickness or chronic bronchitis. Also, air trapping on expiration CT scans, due to the collapse of small airways, could be mediated via such an indirect effect of emphysema. Such a hypothesis could be investigated in depth by contrasting (almost) pure chronic bronchitis and (almost) pure emphysema patients, thereby excluding the overlap between the chronic bronchitis and emphysema phenotype [38].

A recent review on the potential role of sRAGE as biomarker for COPD described several limitations of sRAGE [19], including the lack of a clinically validated assay, leading to a rather wide disparity in absolute values found between studies. The current study used a fully validated sRAGE assay following FDA guidelines [24]. By using this assay, we recently demonstrated that smoking immediately decreases sRAGE values in serum [25, 39]. Particularly in current smokers this may decrease the biomarker function of sRAGE and explain in part the variation observed in the 25% current smokers of our study. A second pitfall described in the review is the potentially confounding effect on sRAGE by a number of comorbidities [19]. We agree that this should be taken into account in larger studies than the current one. Another limitation is the scarce available information on the therapeutic modulation of RAGE signaling in human disease [19]. However, it was shown in mice that overexpression of RAGE induces inflammation and causes airspace enlargement [40], whereas RAGE knockout mice hardly develop cigarette smoke- or elastase-induced emphysema [41, 42], further supporting that RAGE signaling plays an important mechanistic role in emphysema. A further limitation of the potential biomarker role of sRAGE, not described in the abovementioned review, is its inverse relationship with disease activity of COPD, meaning that the plasma levels of sRAGE are decreasing with an increase in disease severity. This inverse relationship is probably due to the fact that free circulating sRAGE acts as a protective decoy-receptor, by binding pro-inflammatory RAGE-ligands and thus avoiding the activation of membrane bound RAGE as well as preventing the homodimerization of RAGE needed for downstream signaling of RAGE. In that perspective, we believe that the biomarker function of sRAGE can be improved by studying sRAGE in the context of its ligands, particularly the ligands that are involved in emphysematous processes.

A strength of our study is that emphysema was visually subtyped into paraseptal and centrilobular emphysema. Although visual scoring is subjective, it was performed by researchers who were blinded to any clinical or functional information, demonstrating high inter-observer agreement, comparable to other observations in literature [43, 44]. Nevertheless, there is a clear unmet need to optimize the radiological characterization of COPD, and particularly the automated quantification of subtypes of emphysema [45]. Furthermore, the plasma sRAGE levels between none, trace, mild and moderate centrilobular emphysema were quite similar, likely because these categories of emphysema are often quite localized and therefore would result in relatively little changes in circulating sRAGE levels.

## Conclusion

In conclusion, this is the first study demonstrating a strong association between sRAGE and the visually scored severity of centrilobular emphysema. In contrast, there was no association with paraseptal emphysema nor with airway wall thickness. This specificity of sRAGE for centrilobular emphysema shows that endotyping of a disease needs careful and meticulous (sub)phenotyping of the disease. Further studies are needed to replicate our finding, also including more severe emphysema patients.

## Data Availability

The datasets generated and/or analyzed during the current study are available in the PASSEL repository under the dataset identifier PASS01670, http://www.peptideatlas.org/PASS/PASS01670.

## Abbreviations

ADAM10: A disintegrin and metalloproteinase domain-containing protein 10
AGER: Advanced glycosylation end-product specific receptor
BMI: Body mass index
CLE: Centrilobular emphysema
COPD: Chronic obstructive pulmonary disease
CT: Computed tomography
FDA: Food and Drug Administration
FEV_1_: Forced expiratory volume in 1 s
FVC: Forced vital capacity
GOLD: Global initiative for chronic obstructive lung disease
HD: Heterogeneity difference
HRCT: High-resolution computed tomography
HU: Hounsfield Units
IPE: Immunoprecipitation in 96-well enzyme-linked immunosorbent assay
LC–MS: Liquid chromatography–mass spectrometry
LLL: Left lower lobe
LUL: Left upper lobe
MMP9: Matrix metallopeptidase 9
NA: Not applicable
ND: Not determined
Perc15: 15Th percentile of the lung density histogram
Pi10: Square root of the wall area at the inner perimeter of a 10 mm diameter airway
PRM: Parametric response mapping
PSE: Paraseptal emphysema
RAGE: Receptor for advanced glycation end-products
RLL: Right lower lobe
RML: Right middle lobe
RUL: Right upper lobe
SD: Standard deviation
SEM: Standard error of the mean
SNP: Single nucleotide polymorphism
sRAGE: Soluble receptor for advanced glycation end-products
%LAA: Percentage of low attenuation areas
%LAA950: Percentage ratio of low-attenuation areas below a threshold of − 950 Hounsfield units
